# Current Guidelines Have Limited Applicability to Patients with Comorbid Conditions: A Systematic Analysis of Evidence-Based Guidelines

**DOI:** 10.1371/journal.pone.0025987

**Published:** 2011-10-20

**Authors:** Marjolein Lugtenberg, Jako S. Burgers, Carolyn Clancy, Gert P. Westert, Eric C. Schneider

**Affiliations:** 1 Scientific Centre for Care and Welfare (Tranzo), Tilburg University, Tilburg, The Netherlands; 2 Scientific Institute for Quality of Healthcare (IQ Healthcare), Radboud University Nijmegen Medical Centre, Nijmegen, The Netherlands; 3 Dutch College of General Practitioners (NHG), Utrecht, The Netherlands; 4 Agency for Healthcare Research and Quality (AHRQ), Rockville, Maryland, United States of America; 5 Harvard School of Public Health, Boston, Massachusetts, United States of America; 6 RAND Corporation, Boston, Massachusetts, United States of America; 7 Division of General Medicine and Primary Care, Brigham and Women's Hospital, Boston, Massachusetts, United States of America; University of Oxford, United Kingdom

## Abstract

**Background:**

Guidelines traditionally focus on the diagnosis and treatment of single diseases. As almost half of the patients with a chronic disease have more than one disease, the applicability of guidelines may be limited. The aim of this study was to assess the extent that guidelines address comorbidity and to assess the supporting evidence of recommendations related to comorbidity.

**Methodology/Principal Findings:**

We conducted a systematic analysis of evidence-based guidelines focusing on four highly prevalent chronic conditions with a high impact on quality of life: chronic obstructive pulmonary disease, depressive disorder, diabetes mellitus type 2, and osteoarthritis. Data were abstracted from each guideline on the extent that comorbidity was addressed (general comments, specific recommendations), the type of comorbidity discussed (concordant, discordant), and the supporting evidence of the comorbidity-related recommendations (level of evidence, translation of evidence). Of the 20 guidelines, 17 (85%) addressed the issue of comorbidity and 14 (70%) provided specific recommendations on comorbidity. In general, the guidelines included few recommendations on patients with comorbidity (mean 3 recommendations per guideline, range 0 to 26). Of the 59 comorbidity-related recommendations provided, 46 (78%) addressed concordant comorbidities, 8 (14%) discordant comorbidities, and for 5 (8%) the type of comorbidity was not specified. The strength of the supporting evidence was moderate for 25% (15/59) and low for 37% (22/59) of the recommendations. In addition, for 73% (43/59) of the recommendations the evidence was not adequately translated into the guidelines.

**Conclusions/Significance:**

Our study showed that the applicability of current evidence-based guidelines to patients with comorbid conditions is limited. Most guidelines do not provide explicit guidance on treatment of patients with comorbidity, particularly for discordant combinations. Guidelines should be more explicit about the applicability of their recommendations to patients with comorbidity. Future clinical trials should also include patients with the most prevalent combinations of chronic conditions.

## Introduction

Traditionally, medical care is focused on the prevention, diagnosis and treatment of single diseases [1]. Most research studies focus on the effectiveness of disease-specific interventions and patients with comorbidity or complex problems are often excluded from clinical trials [2], [3]. In clinical practice, physicians are encouraged to adhere to evidence-based clinical practice guidelines (CPGs), as these are regarded as important tools for quality improvement [4]. In line with both clinical practice and research traditions, most CPGs are disease-oriented documents focusing on the diagnosis and management of single diseases [5].

The emphasis of CPGs on single diseases may be problematic. Almost half of patients with chronic diseases have more than one disease [6], [7]. Managing multiple conditions is more complex than managing single diseases and clinicians may find it challenging to provide optimal care for patients with multiple conditions [8]–[10]. Particularly when conditions are discordant, i.e. if they are not directly related in either their pathogenesis or management and do not share an underlying predisposing factor, patients are more likely to report conflicting instructions and problems with coordination of care [11]–[13].

To the extent that CPGs focus on single diseases, they may offer insufficient guidance to physicians about care for patients with multiple conditions. Lack of applicability of CPGs due to comorbidity may pose an important barrier to guideline adherence among physicians [14], [15]. Moreover, adhering to single disease CPGs in caring for patients with multiple conditions may adversely affect patient safety, if recommended treatments for one condition conflict with those for another condition [16].

Although prior studies suggest that physicians may find it challenging to provide care to patients with comorbidity, there are few systematic assessments of the comorbidity-related content of CPGs, and in particular the quality of the evidence that supports that content. The aim of this study was to explore the applicability of CPGs to patients with comorbidity by assessing the extent to which CPGs on high-prevalence chronic conditions address comorbidity and by assessing the quality of the evidence cited in support of recommendations related to comorbidity.

## Methods

### Data sources

Two publicly-available international databases, the National Guideline Clearinghouse (NGC) and the Guidelines International Network Library (G-I-N),were used to select the guidelines.

### Study selection

#### Selection of chronic conditions

In selecting the conditions, we focused on highly prevalent chronic diseases that have a high impact on quality of life. Both major depressive disorder [17], [18] and diabetes mellitus type 2 [19], [20] are highly prevalent and have been found to have a high impact on quality of life, particularly in combination [17], [21]. We also included chronic obstructive pulmonary disease (COPD) and osteoarthritis, as pain and dyspnea may have a considerable impact on quality of life as well.

### Selection of clinical practice guidelines

Guidelines were included if they:

- included a set of recommendations with an explicit link to their supporting evidence;
- were published in 2005 or later;
- addressed the treatment or management of the selected conditions;
- were published in English;
- were accessible in the public domain.

CPGs were excluded if they focused on a specific subgroup of patients (e.g. pregnant women, children, adolescents, homeless people).

### Data extraction

One of the investigators (ML) abstracted data from the selected CPGs and the abstraction process was checked by a second investigator (JB). Any disagreement was resolved by discussion. General data were retrieved from the CPGs, and more detailed information was collected on the specific recommendations addressing comorbidity and their supporting evidence:

### Guideline

- General characteristics of the guideline: title; organization; country; target group; year of publication; number of pages and references; number of treatment recommendations.
- Characteristics of the guideline related to comorbidity: issue of comorbidity addressed (prevalence data, screening/diagnosing for comorbidity; considering comorbidity in treatment); discussion of patient-centered aspects (such as goals and burden of treatment, incorporating patient preferences), inclusion of specific comorbidity-related treatment recommendations (number and proportion). A recommendation was defined as a statement whose apparent intent is to provide guidance about the advisability of a clinical action [22]. Contra-indications for medication or surgery were not considered as specific comorbidity-related recommendations, if no alternative treatments were provided.

### Recommendation

- Type of recommendation: type of treatment addressed (general treatment, drug therapy, life-style advice, surgery, other); inclusion of patient-centered aspects.
- Number of comorbid conditions addressed;
- Type of comorbidity addressed: concordant or discordant. Concordant conditions were defined as representing the same overall pathophysiological risk profile and being more likely to be the focus of the same disease and self management plan [12]. Discordant treatments are not directly related in either their pathogenesis or management. For each of the included conditions the authors developed a scheme of concordant and discordant comorbidities ( File S1). For diabetes, we did not consider cardiovascular risk factors such as hypertension and hyperlipidemia as concordant conditions but as part of the disease, because adequate management of diabetes is cardiovascular risk management including monitoring blood pressure and lipids.

### Evidence

- Link with underlying evidence described; (yes, no)
- Number of underlying studies;
- Level of evidence of underlying studies: high, moderate, low, not available. As grading systems differ per guideline, we considered the highest level of evidence as high, the lowest level as low, and intermediate levels as moderate.
- Translation of evidence: good, moderate or poor/unclear. Our judgment was based on the directness of the evidence and on whether the strengths and limitations of the evidence were discussed in the guideline. The translation was graded as: ‘good’ if the supporting evidence of the studies focused (at least partly) on the comorbidity part of the recommendation and the strengths and limitations of the supporting evidence were discussed in the guideline; as ‘moderate’ if either the supporting evidence of the studies focused (at least partly) on the comorbidity part of the recommendation or the strengths and limitations of the supporting evidence were discussed in the guideline; and as ‘poor or unclear’ if neither the supporting evidence of the studies focused on the comorbidity part of the recommendation nor were the strengths and limitations of the supporting evidence discussed in the guideline.

## Results

A total of 20 CPGs met our inclusion criteria, having been published in English and in the public domain since 2005 (Table 1). Six of the CPGs addressed COPD, four addressed major depressive disorder, seven addressed diabetes mellitus type 2 and three addressed osteoarthritis.

**Table 1:** Basic characteristics of selected guidelines (N = 20).

Title of guideline	Organization that developed guideline	Country	Year	No. of pages	No. of references
**COPD**					
1. Chronic obstructive pulmonary disease	Singapore Ministry of Health	Singapore	2006	84	155
2. Diagnosis and management of Chronic obstructive pulmonary disease (COPD)	Institute for Clinical Systems Improvement (ICSI)	USA	2009	51	97
3. Diagnosis and management of stable chronic obstructive pulmonary disease: a clinical practice guideline from the American College of Physicians	American College of Physicians	USA	2007	6	54
4. Global strategy for the diagnosis, management, and prevention of chronic obstructive pulmonary disease	Global Initiative for Chronic Obstructive Lung Disease - Disease Specific Society (WHO), National Heart, Lung, and Blood Institute (U.S.)	Several countries	2008	94	435
5. Australian Lung Foundation & The Thoracic Society of Australia and New Zealand - The COPD-X Plan: Australian and New Zealand Guidelines for the management of Chronic Obstructive Pulmonary Disease 2006	New Zealand Guidelines Group (NZGG)	New Zealand	2006	66	243
6. Canadian Thoracic Society Recommendations for Management of Chronic Obstructive Pulmonary Disease, CTS (CA)	Canadian Thoracic Society	Canada	2007	28	366
**DEPPRESIVE DISORDER (MAJOR)**					
7. Major depression in adults in primary care	Institute for Clinical Systems Improvement (ICSI)	USA	2008	84	244
8. Identification of common mental disorders and management of depression in primary care	New Zealand Guidelines Group (NZGG)	New Zealand	2008	188	580
9. Using Second-Generation Antidepressants to Treat Depressive Disorders: A Clinical Practice Guideline from the American College of Physicians	American College of Physicians (ACP)	USA	2008	10	100
10. A. Depression: the treatment and management of depression in adults (update) (CG90)	National Institute for Health and Clinical Excellence (NICE)	United Kingdom	2009	64 (FG = 585)	0 (FG>1000)
**DIABETES MELLITUS TYPE 2**					
11. American Association of Clinical Endocrinologists medical guidelines for clinical practice for the management of diabetes mellitus	American Association of Clinical Endocrinologists, American College of Endocrinology	USA	2007	68	564
12. Diabetes mellitus	Singapore Ministry of Health	Singapore	2006	161	260
13. Diagnosis and management of type 2 diabetes mellitus in adults	Institute for Clinical Systems Improvement (ICSI)	USA	2008	89	126
14. Guidelines on diabetes, pre-diabetes, and cardiovascular diseases	European Society of Cardiology	Several European countries	2007	72	711
15. Standards of medical care in diabetes	American Diabetes Association	USA	2008	43	332
16. National evidence-based guidelines for type 2 diabetes mellitus (Part 1, 3, 4, 5 & 7)	National Health and Medical Research Council (NHMRC)	Australia	2005	928	>1000
17. Type 2 diabetes - the management of type 2 diabetes (partial update)+newer agents (CG87)	National Institute for Health and Clinical Excellence (NICE)	United Kingdom	2009	151 (FG = 259)	0 (FG = 414)
**OSTEOARTHRITIS**					
18. Osteoarthritis of the knees	Singapore Ministry of Health	Singapore	2007	51	91
19. The care and management of osteoarthritis in adults	National Institute for Health and Clinical Excellence (NICE)	United Kingdom	2008	22 (FG = 316)	0 (FG = 386)
20. Ottawa Panel evidence-based clinical practice guidelines for therapeutic exercises and manual therapy in the management of osteoarthritis	Ottawa Panel	Canada	2005	65	178

FG = Full guideline.

Eight CPGs were retrieved from the G-I-N database, six from the NGC database and six were available in both databases. The largest share of these 20 CPGs was produced in the United States (n = 7). Nine CPGs were produced by governmental agencies; five by professional societies and six by other types of organizations. The CPGs were predominantly developed in 2008 (7/20) and in 2007 (5/20).

### Applicability of guidelines to patients with comorbidity

Of the 20 guidelines, 17 (85%) addressed the issue of comorbidity (Table 2). Eight guidelines (40%) provided comorbidity prevalence data, 16 guidelines (80%) recommended screening for comorbid conditions and 17 guidelines (85%) recommended considering comorbidity in treatment. Guidelines on depressive disorder and diabetes mellitus type 2 (100%) more often addressed the issue of comorbidity compared to the guidelines on COPD (83%) and osteoarthritis (33%).

**Table 2:** Characteristics of guidelines in terms of addressing comorbidity (N = 20).

Guidelines	COPD (N = 6)		DEP (N = 4)		DM II (N = 7)		OA (N = 3)		TOTAL (N = 20)	
	N	%	N	%	N	%	N	%	N	%
*Issue of comorbidity addressed*	5	83	4	100	7	100	1	33	17	85
Provision of comorbidity prevalence data	3	50	2	50	2	29	1	33	8	40
Screening/diagnosing for comorbidity	5	83	3	75	7	100	1	33	16	80
Considering comorbidity in treatment	5	83	4	100	7	100	1	33	17	85
Inclusion of patient centered aspects	4	67	3	75	4	57	1	33	12	60
*Includes specific comorbidity-related treatment recommendation(s)*	3	50	4	100	6	86	1	33	14	70
Mean number of recommendations per guideline (range)	0.7	(0–2)	2.3	(1–4)	6.3	(0–26)	0.7	(0–2)	3.0	(0–26)

COPD = Chronic Obstructive Pulmonary Disease; DEP = Major depressive disorder; DM II = Diabetes Mellitus type 2; OA = Osteoarthritis.

Fourteen (70%) guidelines provided specific treatment recommendation for patients with comorbid conditions. The number of recommendations varied from 1 to 26 per guideline, with an average of 3 per guideline. The guidelines on COPD and osteoarthritis provided the fewest numbers of recommendations (0.7 per guideline), whereas the guidelines on diabetes mellitus type 2 included an average of 6.3 comorbidity-related recommendations.

The 20 guidelines provided a total of 59 comorbidity-related treatment recommendations (Table 3). Seventy-eight percent (46/59) of these recommendations addressed concordant comorbidities. Most of the diabetes mellitus type 2 guideline recommendations addressed concordant comorbidities such as coronary artery disease and heart failure. Relative to the other guidelines, the guidelines on depressive disorder included the largest proportion (33%) of recommendations on discordant comorbidities (such as cardiovascular disease). More than 90% of the recommendations were related to one comorbid condition; 10% focused on comorbidities in general and none of the recommendations specified the management of patients with more than one comorbid condition.

**Table 3:** Characteristics of comorbidity-related treatment recommendations (N = 59).

Comorbidity-related treatment recommendations	COPD (N = 4)	DEP (N = 9)	DM II (N = 44)	OA (N = 2)	TOTAL (N = 59)	
	N	N	N	N	N	%
*Type of comorbidity addressed*						
concordant comorbidity	3	5	38	0	46	78
discordant comorbidity	1	3	4	0	8	14
not specified	0	1	2	2	5	8
*Nr of comorbid conditions addressed*						
one comorbid condition	4	8	42	0	54	92
multiple comorbidities	0	0	0	0	0	0
not specified	0	1	2	2	5	8
*Type of recommendation*						
general treatment	0	3	1	0	4	7
drug therapy	1	4	27	0	32	54
life-style advice	0	0	1	1	2	3
surgery	0	0	5	1	6	10
other*	3	2	10	0	15	25
Includes patient centered aspects	0	3	4	0	7	12

COPD = Chronic Obstructive Pulmonary Disease; DEP = Major depressive disorder; DM II = Diabetes Mellitus type 2; OA = Osteoarthritis.

*The category ‘other’ includes: psychological interventions, oxygen therapy, referral, assessment before flying, target levels, risk stratification.

Fifty-four percent of the comorbidity-related recommendations concerned drug therapy (32/59); 25% related to other types of treatment such as psychotherapy or oxygen therapy (15/59). Few recommendations focused on surgery (10%; 6/59) and on life-style advice (3%; 2/59). Twelve percent of the recommendations (7/59) provided specific guidance on patient-centered aspects such as patient preferences, burden of disease and priority setting.

The link between guideline recommendation statements and the supporting evidence was described for 97% of the recommendations (57/59). The number of underlying studies varied between 1 and 12 per recommendation. The level of evidence of the studies was generally weak: 37% of the recommendations (22/59) had a ‘low’ level of evidence; for 25% of the recommendations (15/59) the level of evidence was described as ‘moderate’ (Table 4 and 5).

**Table 4:** Evidence-base of comorbidity-related treatment recommendations (N = 59).

Comorbidity-related treatment recommendations	COPD (N = 4)	DEP (N = 9)	DM II (N = 44)	OA (N = 2)	TOTAL (N = 59)	
	N	N	N	N	N	%
*Number of underlying studies*						
0 or unclear	1	1	7	1	10	17
1–2	3	4	12	0	19	32
3–4	0	3	11	0	14	24
>4	0	1	14	1	16	27
*Level of evidence of the studies*						
high	2	0	14	0	16	27
moderate	1	2	12	0	15	25
low	1	5	16	0	22	37
N.A.	0	2	2	2	6	10
*Translation of evidence*						
good	0	2	14	0	16	27
moderate	3	3	22	0	28	48
poor or unclear	1	4	8	2	15	25

COPD = Chronic Obstructive Pulmonary Disease; DEP = Major depressive disorder; DM II = Diabetes Mellitus type 2; OA = Osteoarthritis.

**Table 5:** Examples of comorbidity-related treatment recommendations with different levels of supporting evidence.

Example of recommendation with moderate level of evidence and good translation of evidence
“Diabetic patients with acute myocardial infarction benefit from a tight glucometabolic control. This may be accomplished by different treatment strategies”
*Level of evidence:* MODERATE (Class IIa; Level B)
*Translation of evidence:* GOOD
“Metabolic support and control: There are several reasons why intensive metabolic control during an acute myocardial infarction should be of benefit [several studies are described ….]. Based on present knowledge, there is reasonable evidence to initiate glucose control by means of insulin infusion in diabetic patients who are admitted for AMIs with significantly elevated blood glucose levels in order to reach normoglycaemia as soon as possible. Patients admitted with relatively normal glucose levels may be handled with oral glucose-lowering agents. In the follow-up, both epidemiological data and recent trials support that continued strict glucose control is beneficial. The therapeutic regime to accomplish this goal may include diet, life styles strategies, oral agents, and insulin (see also section on life style and comprehensive management). Since there is no definite answer to which pharmacological treatment is the best choice, the final decision can be based on decisions by the physician-in-charge in collaboration with the patient. Most importantly, the effect on long-term glucose control has to be followed and the levels should be targeted to be as normal as possible. Several outcome studies with novel agents or regimens are ongoing and will report in the near future.”
*Comment:* Several studies are discussed directly targeting the group of diabetic patients with AMI. The strengths and limitations of the available evidence are clearly discussed and taken into consideration in making the final recommendation.

Example of recommendation with high level of evidence and moderate translation
“Prevent or treat osteoporosis (in patients with COPD)”
*Level of evidence:* HIGH (A)
*Translation of evidence:* MODERATE
“Intervention should be targeted at men and women who are taking more than 15 mg daily of prednisolone or who have several risk factors for osteoporosis and whose BMD is <1.5 standard deviations below the young adult mean (Ref 88). Oral bisphosphonates, particularly risedronate, have been shown to be effective in preventing and treating bone loss in men and women taking corticosteroids (Ref 88, 219). However, most patients in these studies did not have respiratory disease. Selecting patients with COPD who may be at increased risk of osteoporosis is most appropriately done on the basis of conventional risk factors. Further refining of clinical predictors and more evidence for the cost effectiveness of such programs still needs to be resolved before recommendations on a screening strategy in patients with COPD can be made. For more information on prevention and treatment of osteoporosis, see the current Australian guidelines.”
*Comment:* Several studies and their limitations are described, but the studies are not directly focused on patients with respiratory diseases.

Example of recommendation with low level of evidence and good translation
“Treat depressed cardiac patients……….”
*Level of evidence:* LOW (Consensus statement)
*Translation of evidence:* GOOD
“As yet there are no data to support the hypothesis that antidepressant treatment improves cardiac morbidity and mortality (Jiang, 2005 [R]). Nevertheless, consensus opinion is to treat depressed cardiac patients with a safe drug rather than watchful waiting since they would benefit from symptomatic relief of their depressive symptoms and there is a potential improvement in their cardiovascular risk profile (Ballenger, 2001 [R]).”
*Comment:* The evidence (Ballenger JC, Davidson JRT, Lecrubier Y, et al. Consensus statement on depression, anxiety, and cardiovascular disease. *J Clin Psychiatry* 2001) directly applies to the group of comorbid patients. Moreover, they discuss the strengths and limitations of the evidence and take these into account in formulating the recommendation.

For 73% of the recommendations (43/59), the evidence underlying the studies was not adequately translated into the guideline with 48% (28/59) graded as ‘moderate’ and 25% (15/59) as ‘poor or unclear’ (Table 4 and 5). Translation of evidence was rated more frequently as ‘good’ for guidelines on diabetes mellitus type 2 (32% [14/44]) than those on depression (22% [2/9]); none of the guidelines on COPD and osteoarthritis received a ‘good’ rating for evidence translation (Table 4).

## Discussion

Patients with multiple comorbid conditions are very frequently encountered in clinical practice. However, our results suggest that evidence-based guidelines on four relatively prevalent chronic diseases may have limited applicability to patients with comorbid conditions. Most of these guidelines do not provide explicit guidance on treatment of patients with specific combinations of diseases. If comorbidity is addressed in the guidelines, it is often discussed in general; few specific treatment recommendations for patients with comorbid conditions are provided, particularly for discordant combinations. Moreover, the evidence supporting the available comorbidity-related recommendations was generally limited, had moderate to poor quality, and was often not adequately translated into the guidelines.

Among the guidelines in our study that included specific comorbidity-related recommendations, these recommendations were more likely to focus on concordant comorbidities with related treatment plans. We also found that none of the comorbidity-related recommendations specified the preferred action for patients with more than one concurrent condition. These results are consistent with previous American [16] and Australian [23] studies showing that guidelines pay little attention to patients with discordant comorbidities and to patients with multiple chronic conditions. This lack of attention contributes to limiting the applicability of single disease guidelines on patients with chronic diseases as almost one third of them have three or more conditions [24].

An important finding of our study is the limited evidence base that supports comorbidity-related recommendations. If specific recommendations for patients with comorbidity are provided, they are often based on limited evidence that is of moderate or poor quality. In addition, the supporting evidence rarely focuses directly on the groups of patients with comorbid conditions. Furthermore, the limitations of this evidence are not usually described in the guidelines. The failure to describe limitations of evidence in a guideline could give clinicians misplaced confidence in guideline recommendations.

Consistent with previous studies, our findings indicate that the evidence base for patients with multiple chronic conditions is limited [2], [3]. The lack of evidence specific to comorbid conditions may explain the limited attention to comorbidity in the guidelines we studied. If future clinical trials included patients with comorbid conditions, at least for the most common combination of diseases and report the results, this would provide the evidence base that clinical guideline developers need [16], [25].

In light of the general absence of research evidence on patients with multiple conditions, guidelines should be more explicit about the applicability of their recommendations to patients with the most prevalent comorbid conditions and discuss the quality and directness of the evidence for these patients. This explicit approach should replace the implicit assumption that guideline recommendations are applicable to patients with comorbid conditions unless conflicting evidence is available [26], [27].

Our findings indicate that no systematic approach is used by guideline development groups for addressing comorbidity in guidelines. Compared to the guidelines on COPD, depressive disorder, and osteoarthritis, the guidelines on diabetes mellitus type 2 had better reporting of issues of comorbidity. Even for guidelines on the same condition, we found large variation between guidelines in the approach to addressing comorbidity. This applies to all levels of abstraction (guideline, recommendation, evidence). A previous study comparing diabetes guidelines from different countries, also found much variation in the supporting evidence, whereas the recommendations were similar [28]. It would be helpful to develop guidance, as part of a handbook or manual for guideline developers [29], [30] to facilitate and support this process and to create more uniformity. In addition, targeting educational activities to professional societies that do not yet incorporate comorbidity to a large extent in their guidelines might be useful.

The main strength of our study is that we systematically assessed the content of an international sample of evidence-based national and international guidelines in terms of addressing comorbidity. The guidelines included in our study are among the best in the clinical areas of interest and were produced by prominent governmental agencies or professional organizations. Furthermore, by simultaneously assessing the underlying evidence of the comorbidity-related recommendations, we were able to determine whether guidance was provided on treatment of patients with comorbid conditions and also to what extent this guidance was based on high-quality evidence.

Our study has several limitations. First, a limited number of chronic conditions were included in our study. Inclusion of a different set of chronic conditions could have yielded different results. However, we do not expect guidelines on other diseases to be more applicable to patients with multiple conditions than those for the included common conditions. Second, the number of selected guidelines varied between the conditions, with an overrepresentation of diabetes guidelines. This reflects the available number of high-quality guidelines on the selected diseases in the databases. Third, we did not assess all available comorbidity-related evidence for the included chronic conditions, but only the evidence that was described in the guidelines. A systematic search for evidence would be necessary to determine whether the guideline recommendations are based on the *best available* evidence. Future research on the selected conditions could be useful to draw firm conclusions on the availability of evidence for patients with multiple conditions, complementing the findings of our study.

Among a selected set of high-quality current evidence-based guidelines on prevalent chronic diseases, there is limited guidance on treatment of patients with comorbid conditions. Although the issue of comorbidity is recognized by guidelines, very few specific recommendations are provided and these are generally based on limited evidence of low or moderate quality. The supporting evidence often does not focus directly on groups of patients with comorbid conditions and it is rare that guidelines adequately describe the limitations of the evidence. Given the increasing prevalence of patients with multiple chronic diseases, guidelines should at least be explicit and transparent about the applicability of their recommendations to populations of patients with the most common combination of diseases. A guide for guideline developers could facilitate a systematic and uniform approach.

## Supporting Information

File S1 **Classification of concordant and discordant comorbidities.**
(DOC)
