# Supplementary material for: Current Guidelines Have Limited Applicability to Patients with Comorbid Conditions: A Systematic Analysis of Evidence-Based Guidelines
Source: PLoS One. 2011 Oct 20;6(10):e25987. doi: 10.1371/journal.pone.0025987 (PMC3197602; doi:10.1371/journal.pone.0025987)
Supplement: File S1 — Classification of concordant and discordant comorbidities. (DOC) [file pone.0025987.s001.doc]

# Supplementary file 1: Classification of concordant and discordant comorbidities

# COPD

# *Risk factors: Complications:*

Smoking Pulmonary hypertension Respiratory Failure

Conditions identified as concordant with COPD:

Cor pulmonale

Heart failure

Conditions identified as discordant with COPD:

Osteoporosis

Depressive disorder

Obesity

Unstable angina

Myocardial infarction

Arthritis

Atrium fibrillation

**Depressive disorder (major)**

Conditions identified as concordant with Depressive disorder:

Anxiety disorders

Bipolar disorders

Personality disorders

Substance abuse/dependence

Dementia

Chronic pain (syndrome)

Conditions identified as discordant with Depressive disorder:

Cardiovascular diseases

Diabetes

Parkinson’s disease

Cancer

**Diabetes type 2**

*Risk factors for diabetes/ atherosclerotic CVD: Complications:*

Obesity Retinopathy

Hypercholesteroleamia/ dyslipidaemia Nephropathy/ renal

Hypertension disease/ chronic kidney

Smoking disease

Neuropathy

End/Organ damage

Gastroparesis

Conditions identified as concordant with Diabetes type 2:

*Cardiovascular disease (CVD)*

Coronary artery disease (CAD):

- Congestive heart failure

- Myocardial infarction (MI)

- Angina pectoris (AP)

Cerebral infarction,

Transient ischaemic attack (TIA),

Peripheral arterial vascular disease

Aortic aneurysm abdominalis

Conditions identified as discordant with Diabetes type 2:

Chronic low back pain

Prostate cancer/ Benign prostatic hypertrophy

Asthma

Depressive disorder

COPD

Gout

Atrium Fibrillation

**Osteoarthritis**

*Risk factors:*

Obesity

Conditions identified as concordant with Osteoarthritis:

Rheumatic arthritis

Low back pain

Conditions identified as discordant with Osteoarthritis:

Atherosclerotic cardiovascular diseases (including risk factors):

Ischaemic heart disease

Stroke

Congestive heart failure

Hypertension

Smoking

Hyperlipideamia

Diabetes

Peripheral arterial disease

Chronic pain syndrome

Depressive disorder
